# Electrochemical Performance of Metal-Free Carbon-Based Catalysts from Different Hydrothermal Carbonization Treatments for Oxygen Reduction Reaction

**DOI:** 10.3390/nano14020173

**Published:** 2024-01-12

**Authors:** Aldo Girimonte, Andrea Stefani, Clara Mucci, Roberto Giovanardi, Andrea Marchetti, Massimo Innocenti, Claudio Fontanesi

**Affiliations:** 1 Department of Engineering, DIEF, University of Modena and Reggio Emilia, via Vivarelli 10, 41125 Modena, Italy; aldo.girimonte@unimore.it (A.G.); 165688@studenti.unimore.it (C.M.); roberto.giovanardi@unimore.it (R.G.); 2 Department of Physics, FIM, University of Modena and Reggio Emilia, via Campi 213, 41125 Modena, Italy; andrea.stefani@unimore.it; 3 Department of Chemical and Geological Science, DSCG, University of Modena and Reggio Emilia, via Campi 103, 41125 Modena, Italy; andrea.marchetti@unimore.it; 4 Department of Chemistry, “Ugo Schiff”, University of Firenze, via della Lastruccia 3, 50019 Sesto Fiorentino, Italy; m.innocenti@unifi.it; 5 National Interuniversity Consortium of Materials Science and Technology (INSTM), via G. Giusti 9, 50121 Firenze, Italy

**Keywords:** ORR catalysts, hydrothermal treatment, glucose, nanostructures

## Abstract

This research investigates the difference between products obtained through two hydrothermal carbonization treatments. Our aim is to synthesize metal-free, carbon-based catalysts for the oxygen reduction reaction (ORR) to serve as efficient and cost-effective alternatives to platinum-based catalysts. Catalysts synthesized using the traditional hydrothermal approach exhibit a higher electrocatalytic activity for ORR in alkaline media, despite their more energy-intensive production process. The superior performance is attributed to differences in the particle morphology and the chemical composition of the particle surfaces. The presence of functional groups on the surfaces of catalysts obtained via a traditional approach significantly enhances ORR activity by facilitating deprotonation reactions in an alkaline environment. Our research aims to provide a reference for future investigations, shifting the focus to the fine-tuning of surface chemical compositions and morphologies of metal-free catalysts to enhance ORR activity.

## 1. Introduction

The oxygen reduction reaction (ORR) has gained significant attention in recent years. ORR is the electrochemical process by which molecular oxygen is reduced, typically forming water in acidic environment or hydroxide ions in alkaline environments. This reaction assumes a significant role in energy conversion devices, including fuel cells, metal–air batteries and electrolyzers [1,2]. As the global push for sustainable and renewable energy solutions increases, the efficient and cost-effective conversion and storage of energy has become a fundamental topic for scientific researches. 

The ORR is a sluggish chemical process where precious metals are used as catalysts,, with platinum (Pt) being the most effective [3,4]. However, the prohibitive cost, scarcity, and sustainability associated with Pt and other noble metals hinders the research on alternatives. Carbon-based metal-free catalysts have shown promising properties in alkaline environments [5,6,7], especially if focusing on the prospect of obtaining a high performance at a fraction of the cost and with widely available resources. These materials also show some obstacles on the path to commercial utilization, with issues related mainly to their long-term stability, selectivity, and kinetics, often in contrast to their noble metal counterparts [8]. Furthermore, the wide variety of carbon materials, from graphene to carbon nanotubes to amorphous carbons, means that a one-size-fits-all approach is usually not possible [9,10,11]. Please note that the use of polymeric/macromolecular organic systems as working electrodes [12,13,14], also employing chiral architectures [15] in electrochemical experiments, is the subject of great interest. In particular, chiral systems proved to be particularly effective concerning the electrochemical processes involving oxygen, in both cases of oxidation and reduction, OER and ORR, respectively [16,17,18]. Within this picture, the use of glucose to synthesize chiral polymeric/macromolecular architectures appears of particular interest. 

A particularly promising strategy to obtain catalysts with a high carbon content and controllable nanostructures is the hydrothermal carbonization technique (HTC). HTC is based on the treatment of aqueous solutions of both organic and inorganic precursors at elevated temperatures and pressures, and allows for the fabrication of carbon materials with highly tunable properties by adjusting parameters like temperature, time, and precursor composition. Some interesting papers have addressed, highlighting the efficacy of HTC-derived carbon materials [17,19,20]. Hydrochar, i.e., the carbonaceous material developed by the HTC process, can be produced using different raw materials, including some monosaccharides or more complex organic chemical compounds [21]. In the case of glucose, the hydrothermal process performed in aqueous solution leads to the dehydration of sugar monomers, which are transformed into furan structures that are able to develop polycondensation reactions by releasing water molecules. The conditions of the applied heat treatment can change the carbon content and structure of the resulting molecule, affecting its chemical properties and degree of reactivity. However, thanks to these characteristics, it is possible to obtain systems in which each molecule is characterized by an internal main structure, composed of bonds between carbon atoms and alcohol groups on the outermost part. While internal carbon structures can be useful in various applications, especially due to the possibility of the intercalation of other atoms within them, for energy purposes, the external groups of the molecular chain are able to deproton more easily in an alkaline environment. Therefore, they can more rapidly generate functional groups that are active as oxygen radicals capable of catalyzing ORR.

Traditional HTC (T-HTC), conducted using standard ovens as an energy source, while effective, is often energy-intensive, but an alternative path can be identified in Microwave-assisted HTC (MW-HTC), promising significant reductions in energy consumption and process time [22]. A comprehensive comparison of these synthesis methods, evaluating aspects ranging from the energy efficiency of the production process to catalyst performance, has been missing in recent studies. Therefore, in our research, we addressed this gap, aiming to underline both the advantages and drawbacks of products obtained through Traditional and Microwave-assisted HTCs.

## 2. Materials and Methods

### 2.1. Reagents

The initial solutions were prepared using D-(+)-Glucose (≥99.5%, Sigma-Aldrich, St. Louis, MO, USA) as the solute and reagent grade Milli-Q (Millipore, Burlington, MA, USA) water as the solvent. Potassium hydroxyde KOH white pellets (≥85%, white pellets, Sigma-Aldrich), poly(vinylidene fluoride) PVDF (average Mw ~534,000 by GPC, powder, Sigma-Aldrich), and 1-Methyl-2-pyrrolidone NMP (99%, Sigma-Aldrich) were used in the characterization stages.

### 2.2. Experimental

#### 2.2.1. T-HTC

In the conventional hydrothermal method (T-HTC), the synthesis starts with the preparation of a 1 M D-(+)-glucose aqueous solution, which is poured in a stainless steel autoclave that is internally equipped with a Teflon liner filling up to 60% of the total volume. The autoclave is then sealed and positioned within a controlled oven using convection heating. The synthesis is carried out with a set temperature of 190 °C, with a isothermal of 15 h. Upon conclusion of the reaction, a filtration procedure is used to separate the dark precipitate. After this step, the solid precipitate is dried at a temperature of 70 °C for a minimum duration of 24 h. The conditions imposed in this synthesis process were defined on the basis of experimental results previously reported in the literature. The sythesis temperature was selected as the lower limit reasonably expected to transform glucose monomers, thus generating the carbonaceous particles. The time taken for the development of the process can be increased or decreased according to the chosen temperature: in our case, a sufficient interval of time was considered to ensure the development of particles with a high carbon content and avoid excessive energy dispersion.

#### 2.2.2. MW-HTC

For this synthesis, aqueous solutions of D-(+)-glucose were formulated with varying molar concentrations: 0.1 M, 0.3 M, 0.5 M and 1 M. Utilizing the capabilities of the machine (model “Milestone ultraWAVE”), which can allow for simultaneous hydrothermal treatments, 20 mL of each prepared solution was poured into individual quartz containers, and each container was then positioned within a vessel containing 120 mL of tap water. All solutions were inserted into the machine’s housing and hermetically sealed. The machine was connected to nitrogen gas (N_2_) and pressurized to 31.5 bar. The synthetic strategy was based on procedures described by [22,23,24] to obtain optimal results. The machine’s operational protocol involves achieving a target temperature within a 15 min cycle, followed by the synthesis phase, where it sustains an internal temperature of 190 °C and pressure of 120 bar for a duration of 30 min [22,23,24]. After complete system cooldown, the resultant solutions are extracted. 

The phase separation in this method faced more challenges than the conventional HTC synthesis, as filtration was ineffective due to the apparent small size of the precipitate, which remains inseparable with a very low amount of residue. To address this, the samples undergo a centrifugation at 4500 rpm for 20 min that facilitates the segregation of synthesized particles. Nonetheless, this separation technique was only effective for concentrations exceeding 0.5 M, while concentrations below this threshold yield minimal material residues that cannot be used for electrochemical or physical characterization. Subsequently the obtained particles undergo a drying process at 70 °C for 24 h.

#### 2.2.3. Characterization 

An initial physical characterization of the separated particles was conducted using SEM microscopy. The 1 M concentration samples were examined, focusing on particles derived from MW-HTC in comparison to those from T-HTC. The 1 M concentration was selected because these samples were the only ones effectively separated in usable amounts and also had a comparable precursor solution concentration. 

To evaluate the ORR-catalytic capabilities of particles derived from diverse glucose syntheses, we conducted electrochemical tests using a rotating disk electrode (RDE), Autolab, connected to an electrochemical cell via a potentiostat, PGSTAT 128N Autolab (Metrohm Italiana Srl, Origgio (VA), Italy). The electrode construction was based on Au substrate, and prior to drop-casting, each substrate is cleaned, starting from a polishing step using a porous disk saturated with a fine Al_2_O_3_ ceramic oxide suspension, followed by a gentle flame annealing process with ethanol flame. Solutions containing the synthesized particles were prepared and subsequently drop-casted on the Au electrode’s surface using a controlled volume micropipette for mass loading control. The drop-casting solution comprised glucose nanospheres suspended in 5% PVDF solution in NMP, and the obtained wet electrode was treated through a vacuum-drying process at 120 °C to eliminate solvent traces, achieving a final mass loading of 5 mg/cm^2^.

The dried electrode was used to build the electrochemical cell, serving as the working electrode (WE) connected to the RDE, which determines its rotational velocity. The setup also incorporated an Ag/AgCl wire as the reference electrode (RE) and a Pt wire as the counter electrode (CE). Experiments were carried out in a 0.1 M KOH aqueous solution, varying the WE rotation speed (500, 1000, 1500, 2000, 2500, and 3000 rpm). Simultaneously, either O_2_ or Ar was fluxed into the solution to achieve oxygen-saturated or oxygen-depleted conditions, respectively. Several Linear Sweep Voltammetry (LSV) tests were conducted on the samples, with each test incrementally elevating the RDE’s rotational speed. A comparative analysis of the LSV curves derived from the 1 M D-(+)-glucose precursor was performed to highlight the impact of a polycondensation process via traditional hydrothermal treatment compared to that usin microwave-assisted approaches.

The conductive ability of the nanospheres was probed by measuring impedance spectra using a CHInstruments 660 potentiostat (CH Instruments, Inc., Austin, TX, USA). The particles were confined between two gold electrodes of 1.5 mm diameter, facing parallel to each other, and placed at 1 mm distance (thus the total volume sampled by impedance measurements was of 7.07 mm^3^). Impedance spectra were recorded for the row of dry particles (the results are reported in Figure S1 in the Supporting Information) and for an “ink” obtained by mixing the glucose nanospheres with a saturated KCl aqueous solution in a 2-to-1 mass ratio. Impedance spectra were recorded using a sinusoidal waveform in the 1 Hz to 100 kHz frequency range, with zero bias, at a 0.05 V peak-to-peak amplitude.

All the electrochemical measurements were carried out at a constant temperature of 25 ± 0.1 °C in a thermostated electrochemical cell.

To further characterize the products synthesized via conventional and microwave-assisted hydrothermal carbonization methods, Fourier transform infrared spectroscopy (FTIR) analysis was conducted. For this purpose, a Vertex 70 FTIR spectrophotometer (Bruker Corporation, Milano (MI), Italy) was used, with a detector kept under control by a liquid nitrogen cooling system, working in attenuated total reflection (ATR) mode with air flow inside the system.

This technique allows for the analysis of solid samples by applying a relatively simple preparation, placing them directly in contact with a component with a high refractive index. The light beam generated by the source is passed through the sample and the optical element, with a series of reflections that allow for the instrument to develop an IR spectrum of the sample. By exploiting the characteristics of the evanescent wave the detector is able to generate curves as a functions of wavelength and transmittance; these are subsequently expressed in the final graph, with respect to absorbance, to facilitate the reading of the graph.

The experiment was conducted by first creating a “blank” IR spectrum of the ATR system, in order to obtain an appropriate baseline. Subsequently, the IR spectra of three different solid samples were obtained:

1. Pure glucose (D-(+)-glucose);
2. Carbonaceous nanostructures obtained from 1 M glucose solution using hydrothermal carbonization (T-HTC);
3. Carbon nanospheres acquired from 1 M glucose solution by microwave-assisted hydrothermal carbonization (MW-HTC).

## 3. Results and Discussion

### 3.1. Synthesis Processes

Following the conclusion of the synthesis, our initial step was a visual qualitative inspection of the obtained products. The wet product suspension resulting from the T-HTC exhibited a gelatinous consistency, resembling caramel in terms of its dark color. This property can be attributed to the emergence of insoluble particles obtained from glucose polycondensation. These synthesis products, after being subjected to filtration and subsequent drying, transformed into dark powders, which were then used for further characterization. Upon visual examination of the MW-HTC process results, it was observed that the clear solutions evolved into dark brown suspensions, with darker colors proportional to the precursor concentration. Centrifugation yielded a wet precipitate with a gel-like consistency, which allowed for the separation and washing of particles from suspension with precursor under 0.5 M. We further observed that, despite a drying period exceeding 24 h, the particle obtained from the centrifugation of MW-HTC retained a caramel-like consistency. This contrasted with the products obtained via the T-HTC, which were easily manipulated and transformed into dry powders for subsequent experimental procedures.

Our hypothesis attributes these behaviors of the synthesis products to the growth phase at which particles are extracted during polycondensation, which, in turn, influences their morphology and surface chemistry. Various mechanisms have been postulated in several articles [19,23,25] for the polycondensation of carbohydrates, and the possible critical phases for D-(+)-glucose can be outlined as the following:

1. **Initiation**: D-(+)-glucose undergoes hydrolysis in hydrothermal conditions (elevated solvent temperatures at the vessel’s high pressures), driven by hydronium ions from the autoionization of water.
2. **Production of Soluble Derivatives**: The advancing hydrolysis yields different soluble derivatives and various acids. Among these products are fructose, furfurals (such as 5-hydroxymethylfurfural (5 HMF)), carboxylic acids (formic, acetic, levulinic, etc.), and aldehydes (formic, acetic, etc.), while H_2_, CO_2_, CO, and various light hydrocarbons (methane, ethane, propane, etc.) can be distinguished in the gaseous phase. The resulting acids, especially those deriving from monosaccharide degradation, serve dual purposes, as they catalyze further reactions and become foundational elements during the latter microstructures’ growth.
3. **Polymerization**: The soluble derivatives enter polymerization reactions through mechanisms like intermolecular dehydration and aldol condensation. Concurrently, there is potential for these polymers to undergo aromatization, resulting in aromatic clusters. When these clusters attain a saturation threshold, they trigger a nucleation event.
4. **Progressive Growth and Settling**: The originating nuclei undergo growth driven by the dehydration of aromatic products. As they grow, their solubility diminishes, making them increasingly hydrophobic and leading them to precipitate.
5. **Particle Clustering**: These hydrophobic particles tend to cluster together, pushing to minimize surface energy. During this phase, carbon-rich spheres separate from the hydrothermal water. The latter development is accentuated by high-temperature processes and alkaline catalysts, while being inhibited by the high concentration of glucose. Once their growth reaches a plateau, the outer surfaces of these microstructures exhibit reactive functional groups, while the inner core is composed of more stable carbon-rich groups.

Variables such as the precursor solution concentration, the reaction temperature and the duration demonstrated a pronounced effect on the product [5,6,22]. Elevated reactant concentrations amplify the polymerization process, yielding uniformly shaped microspheres, while higher temperatures and longer durations lean towards higher degrees of carbonization, resulting in nanospheres of irregular geometries and a reduced presence of surface-oxygen-rich functional groups [26].

We hypothesize that the difference in tackiness exhibited by the samples from the two methods can be traced back to variations in their surface chemistry, particle size, hydrophobicity and related hygroscopicity. 

In fact, the hygroscopic nature of particles could be linked to their size and hydrophobic characteristics. Smaller particles, with larger surface-to-volume ratios, offer more active sites for moisture adsorption, rendering them inherently more hygroscopic than their larger counterparts. This heightened hygroscopicity is further influenced by the density of functional groups present on the particle’s surface. The more surface area is available, the higher the specific density of hydrophilic functional groups, which can attract and retain water molecules. This difference might be amplified by the potentially synergistic effects of the microwaves on the HTC process, which could result in a lesser degree of carbonization. The microwave-assisted method is renowned for its rapid and uniform energy distribution to reactants, achieved through induction of the oscillation of polar groups like hydroxyl and carbonyl, resulting in the generation of heat. The possibility that certain surface groups formed during glucose polycondensation act as focal points for this energy transfer could lead to localized changes in the reaction kinetics and surface affinities of the products. To understand the products’ properties and their relationship with morphological features, the glucose nanospheres were characterized via SEM imaging, vibrational infrared, FTIR, spectroscopy, and impedance spectroscopy.

### 3.2. Physical Characterization

**Figure 1:**
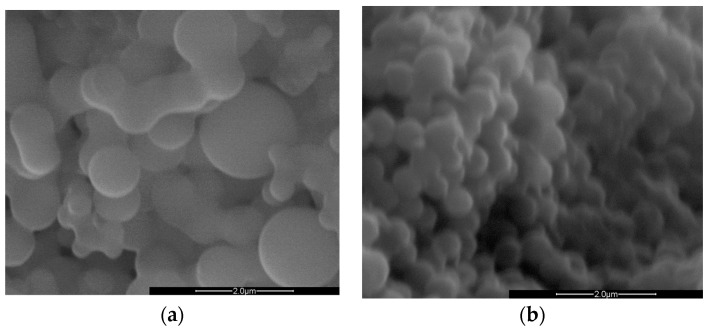
SEM images of carbon nanosphere derived from HTC treatment of D-(+)-glucose (1 M) obtained by (**a**) T-HTC and (**b**) MW-HTC.

We turned to SEM microscopy to visually assess the morphology of the synthesized particles. The SEM images, presented in Figure 1, sustain our observations regarding the differences in particle sizes resulting from the two synthesis methods. In Figure 1a, particles synthesized via T-HTC are shown. Upon first examination, a marked lack of uniformity in particle size was evident in the samples produced by T-HTC. Additionally, several particles are seen in the process of neck formation, an indication of agglomeration during growth leading to larger particle sizes. In contrast, Figure 1b shows particles synthesized via MW-HTC. Here, the size distribution is uniform across the sample, with no significant neck formation.

An image analysis was employed to define the particle size distribution, and the results are reported in Table 1. The particles derived from MW-HTC exhibit a narrower size distribution, while those synthesized by traditional methods display a broader range of sizes. These findings support our hypothesis that MW-HTC leads to a more controlled and uniform particle growth, which, in turn, defines the surface properties.

### 3.3. Electrochemical Characterization

**Figure 2:**
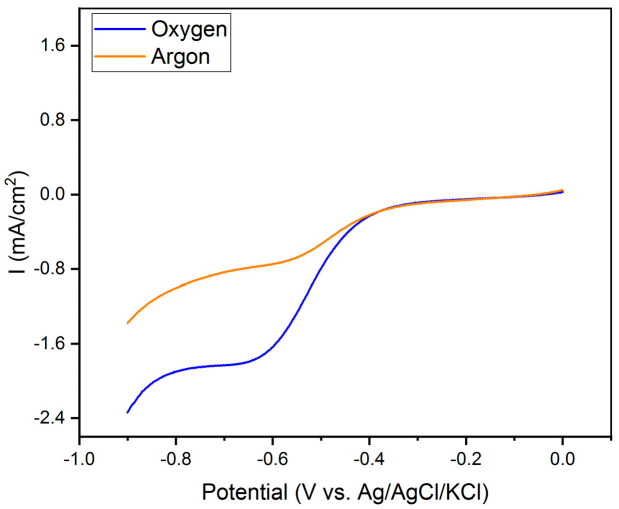
LSV of bare Au electrode as blank curves in de-oxygenated and oxygen saturated in 0.1 M KOH, stationary WE, 0.025 V s^−1^ the potential scan rate.

An electrochemical characterization of the synthesized products was carried out, with the initial preparation and testing of our experimental setup under two distinct conditions to establish a baseline for comparison. The initial trials were conducted with a gold (Au) bare electrode immersed in a 0.1 M KOH solution. This system was purged with argon for a minimum of one hour to ensure low oxygen content. Subsequently, we replicated the experimental setup but altered the environment by saturating the electrolyte with air, thereby introducing a substantial oxygen presence. The outcomes of these experiments are illustrated in Figure 2.

It is evident that no significant reduction current manifests until reaching a potential onset near −0.3 V for both scenarios. At this potential, we observe a reduction peak, which is attributed to the oxygen reduction reaction (ORR). For the sake of comparison, CVs were recorded purging Ar, i.e., reducing the oxygen concentration as much as possible, and in oxygen-saturated solution; please refer to Figure 2 for a comparison. After 10 min argon purging, a residual amount of oxygen remains in the deaerated solution, definitely less than that remaining in the air-saturated one (the current of the CV curves at −0.6 V, Figure 2, corresponding to the Ar purged solution, the red curve, is about 2.25 larger than the oxygen-saturated one, the blue curve). Further exploration into the voltage window reveals an additional onset of reduction beginning at approximately −0.8 V, which we ascribe to the evolution of hydrogen gas (HER), in aligment with the thermodynamic potentials associated with this reaction in an alkaline environment. These observations enabled us to establish a potential window for the investigation of ORR in our system, from −0.1 to −0.7 V, when applying the catalyst as a WE.

**Figure 3:**
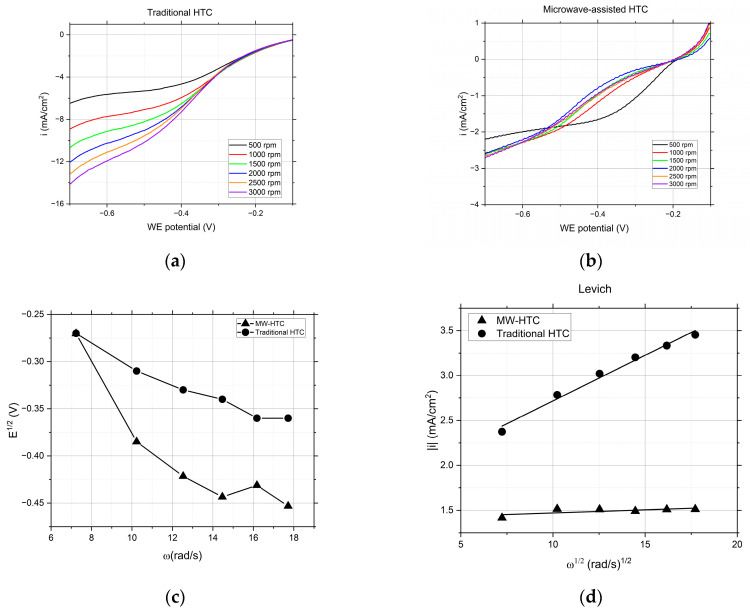
(**a**) LSV of T-HTC catalyst for each of the considered rotation speeds. (**b**) LSV of MW-HTC catalyst for each of the considered rotation speeds. (**c**) Comparison of half wave potentials. (**d**) Comparison of Levich plots.

In Figure 3a, the catalysts derived from T-HTC displayed the expected behavior for ORR. The onset potential for ORR increased compared to the blank test, affirming the catalytic advantage of the catalysts over simple gold substrates. Moreover, as the RDE’s rotation speed was increased, a corresponding rise in limiting current was observed, suggesting the enhanced mass transport of oxygen to the electrode surface. The particles synthesized by MW-HTC exhibited a worse response during the RDE testing, showing only a marginal enhancement of the limiting current during an increase in the rotation speed. This behavior was further analyzed, and the data extracted for the half-wave potential and limiting current are presented in the Levich plot in Figure 3d, offering a visual representation of the rotation-dependent behavior. While the T-HTC particles maintain a pronounced dependency on the rotation rate, with a slope of −1.52 × 10^−5^ A (rad/s)^−1/2^, indicating expected Levich behavior and good electrochemical performance through ORR, the MW-HTC particles show a flatter slope and reduced current values, with only −5.10 × 10^−7^ A (rad/s)^−1/2^. A steeper Levich plot slope indicates a more efficient process, suggesting that the oxygen molecules are more readily reduced at the electrode surface in the T-HTC samples. T-HTC particles outperform MW-HTC in terms of ORR efficiency, so there is a distinct electrochemical phenomenon at play that influences the reactivity and catalytic properties of the ORR on the particle surfaces. 

To estimate the effective area on the surface of the electrode where the reactions for both T-HTC and MW-HTC particles take place, Levich’s equation (Equation (1)) was applied from the experimental data that were obtained:

(1) $$i_{l}=0.62\times n\times F\times A\times D^{2/3}\times \omega^{1/2}\times C_{0}\times \nu^{-1/6}$$

where the number of electrons exchanged *n* is equal to 1, *F* is Faraday’s constant, *A* is the area of the electrode to be determined; the diffusion coefficient of Oxygen *D* results in aqueous solution at 25 °C equal to 0.00242 mm^2^/s, *C*_0_ is the concentration of oxygen in the bulk of the solution equal to 1.22 × 10^−9^ mol/mm^3^, while the kinematic viscosity *υ* is equal to 0.9132 mm^2^/s. Levich’s equation was used in this case based on the assumption of studying a system during steady-state conditions, simplified through hypotheses relative to the values of the above-mentioned constants obtained through literary research. This was because the aim of the test is to demonstrate that the rotational speed variation in the RDE electrode, which influences the increase in current values, is also able to influence the magnitude of the interphase surface affected by the chemical reactions that take place in electrolyte solution. An elaboration of the RDE voltammetric results shown in Figure 3a,b is listed in Table 2. For the sake of comparison, RDE is elaborated on the basis of Equation (1) at two constant potential values: −0.55 and −0.75 V. Indeed, the effective area of T-HTC particles is definitively larger; compare Table 2. The T-HTC effective area is a slightly larger if calculated at −0.7 V, an effect that is likely due to the larger current values in the −0.7 V range, and it is very likely that, at a larger potential, the electrolyte solution is able to more effectively (more effective intergrain penetration) penetrate the nanosphere network. As a result, the effective area is slightly larger.

The calculation was carried out considering the values of angular velocity of the electrode ω within a range of [500; 3000] rpm, and current values relative to the potentials −0.55 V and −0.7 V, which represent the reference extremes of the potential range of interest. The values of the calculated effective area, A, are shown in Table 2:

Our hypothesis is that the MW-HTC particles might facilitate an alternative reduction reaction, concurrent with ORR, in the active material itself. This side reaction could be due to alterations in the active sites on the material’s surface, thus limiting the expected increase in ORR current with higher rotation speeds. 

To investigate these chemical differences, infrared (IR) spectroscopy analyses were conducted. By examining the IR spectra, we can identify the presence and density of various functional groups that contribute to the electrocatalytic activity, such as hydroxyl, carbonyl, and carboxylic groups. 

### 3.4. Infrared Spectroscopic Analysis

**Figure 4:**
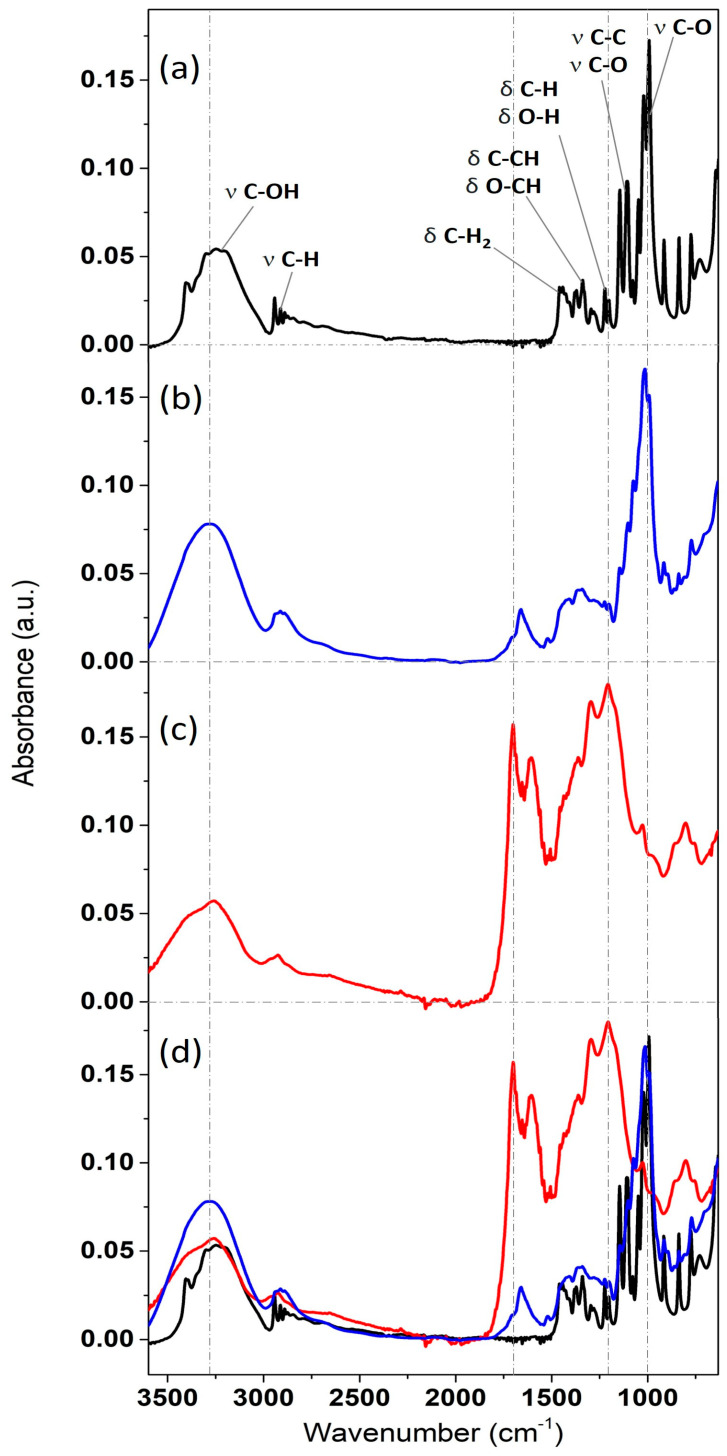
IR spectra of three different samples: (**a**) D-(+)-glucose (black line) (**b**) MW-HTC (blue line) (**c**) T-HTC (red line) and (**d**) overlapping.

Figure 4 displays the IR spectra of the carbon-based particles synthesized both via the slow T-HTC and the more rapid MW-HTC. These are compared with the spectrum of pure D-glucose, represented by the black trace (panel a).

The IR bands of the D-(+)-glucose reagent are in perfect agreement with the scientific literature [27]. Proceeding with decreasing energy, the broad absorption peak extending from 3500 to 3000 cm^−1^ is assigned to the C-O stretching of alcohol groups [28]. The breadth of the structure is due to the presence of signals from both the four secondary alcohol groups and the primary alcohol group (linked to the methylene group). The signals at 2900 cm^−1^ are ascribed to C-H and C-H_2_ stretching vibrations (with the latter related to the methylene group). The bands between 1480 and 1400 cm^−1^ are associated with CH_2_ and C=O bending (scissoring, δ) [29]. The region from 1380 to 1300 cm^−1^ refers to scissoring motions involving carbon, especially C-CH and O-CH. The two bands between 1230 and 1185 cm^−1^ are attributed to CH and OH bending, while, subsequently, a series of high-intensity peaks can be assigned to C-O and C-C stretching vibrations. In particular, the C-O stretching vibrations of up to around 1050 cm^−1^ are related to the four secondary alcohol groups, while the high-intensity peak dominating the spectrum at 1020 cm^−1^ is assigned to the C-O of the primary alcohol group [30]. 

In comparison to what has been presented, the spectra of the heat-treated particles are intensity-normalized to the C-H stretching band at 2900 cm^−1^, since these bonds have only a marginal influence on the polycondensation reaction. This procedure leads to the subsequent natural coincidence in the absorption of the CH_2_ band at 600 cm^−1^. The IR spectrum related to the MW-HTC nanospheres (b panel, blue trace) exhibits a glucose-like character (main band at 1000 cm^−1^ associated with the C-O stretching of primary alcohols, although slightly lower in intensity compared to the black line). However, it also highlights new structures at 1650 cm^−1^, indicative of water incorporation during the heat treatment and the development of new carbonyl (C=O) structures, as well as traces of aromatics (aromatic C-C at 1600–1500 cm^−1^). Furthermore, the increased relative intensity of structures between 1400 and 1150 cm^−1^ suggests polymerization into various high-molecular-weight components through glucose caramelization, with these new structures coexisting with trace amounts of unreacted glucose [19].

The differences between the IR spectrum of MW-HTC particles and that of glucose monomers are accentuated in the case of the T-HTC nanospheres IR spectrum (panel c, red trace). In this case, the band at 1000 cm^−1^ has a much lower relative intensity, and the spectrum is dominated by complex new structures, as evidenced by the high relative intensity of bands at 1700, 1615, 1280, and 1180 cm^−1^. This is a clear fingerprint of the carbon network cross-linking [31], also involving aromatic species and presumably numerous lateral O-H side groups (note the difference between O-H stretching and bending signals in the spectrum of panel c) [32].

### 3.5. Electrochemical Impedance Spectra

**Figure 5:**
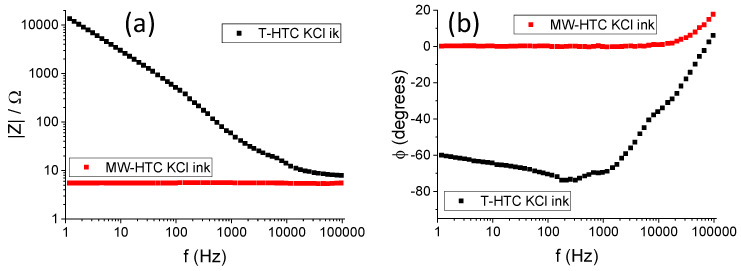
Electrochemical impedance spectra (EIS), Bode plot, for both the T-HTC and MW-HCT particles ink. (**a**) Impedence trend as a function of frequency, (**b**) Fase trend as a function of frequency.

Figure 5 shows the electrochemical impedance spectra of MW-HTC and T-HTC nanospheres dispersed in a KCl 0.1 M solution, at a 2:1 mass ratio, to yield an HTC/KCl ink. The latter was immobilized between two gold surfaces of 3 mm diameter, placed at 1 mm distance. 

Interestingly, the nanospheres obtained via microwave synthesis appear to be intrinsically more conductive with respect to the hydrothermal ones, at least concerning their bulk conductive properties. However, probably, the MW-HTC particles end up in a more sluggish impermeable layer, which, in the end fails, to yield the same catalytic properties for the hydrothermal synthesized particles. Anyhow, please note that in the low-frequency limit, the absolute value of the impedance for the T-HTC sample is around 10 kΩ; although large, this provides a clear indication of the catalytic properties of the hydrothermal nanospheres compared to the catalytic efficiency (which is definitively less) of the particles obtained using microwave-assisted synthesis. 

## 4. Conclusions

The FTIR characterization of the carbonaceous nanoparticles aligns with the structural and electrochemical analysis obtained from SEM and electrochemical experiments, as well as with the impedance results. The morphologies and surface chemistries resulting from the two different thermal treatments have been proved to be a key factor influencing the efficacy of the oxygen reduction reaction (ORR). The microwave-assisted hydrothermal carbonization (MW-HTC) method, despite its rapid processing capability, yields products with a surface chemistry and morphology that are less conducive to ORR compared to those produced by the conventional hydrothermal carbonization (T-HTC) method. The T-HTC method facilitates a higher degree of carbonization, which appears to enhance the catalyst’s effect on ORR. While the MW-HTC offers the advantage of a speedier process, our study suggests that the T-HTC produces products with a superior ORR performance due to a beneficial combination of surface chemistry and structural morphology. The lower reactivity of the carbonaceous particles developed with MW-HTC implies that the method is less effective. The current experiment conditions resulted in an unsuccessful synthesis attempt in the case of MW-HTC heat treatment. This result could, however, be improved by altering process parameters such as the time or temperature. Increasing these parameters in future experiments could lead to a better combination of process conditions, which could promote the development of high-performance structures.

Therefore, this research highlights the importance of considering how different synthesis methods affect the final product’s ability to catalyse the ORR, explaining the need to fine-tune the synthesis process to improve the performance of metal-free carbon catalysts.

Eventually, please note that our particles, with suitable different experimental conditions, hold promise for effective exploitation in catalysing the ORR and/or OER processes. These are of pivotal importance in a number of scientific and technological fields, like water-splitting for hydrogen production, and fuel cell batteries [17]. Furthermore, recent studies demonstrate the fundamental role of chiralized surfaces concerning the relatioship between ORR/spin/anaesthesia [18].

## Figures and Tables

**Table 1:** Results obtained from SEM image analysis for particle surface and form factor measurement.

	Traditional HTC	MW-HTC
Surface average (μm^2^)	4.46	1.57
Sigma surface (μm^2^)	3.70	0.40
Form factor average	1.05	1.06
Sigma Form factor	0.09	0.05

**Table 2:** Electroactive effective surface area of electrodes functionalized with our T-HTC and MW-HTC carbon nanospheres, calculated using Equation (1); voltammetric data shown in Figure 3a,b. Two values of effective area are reported: at −0.55 V and −0.7 V. See the text for a detailed discussion.

Effective Area [mm^2^]			
T-HTC at −0.55 V	T-HTC At −0.7 V	MW-HTC at −0.55 V	MW-HTC at −0.7 V
3.32 × 10^4^	4.58 × 10^4^	2.63 × 10^2^	2.54 × 10^3^

## Data Availability

Data are contained within the article and Supplementary Materials.

## Supplementary Materials

The following supporting information can be downloaded at: https://www.mdpi.com/article/10.3390/nano14020173/s1, Figure S1. Impedace spectra recorded in the 100 kHz to 1 Hz frequecy range, Bode representation, are here reported for the glucose nanospheres in the dry state. (a) sets out the impedance absolute value as a function of the frequency concerning the particles obtained via traditional hydrothermal synthetic route. (b) sets out the phase as a function of the frequency. (c) sets out the impedance absolute value as a function of the frequency concerning the particles obtained via microwave synthetic route. (d) sets out the phase as a function of the frequency.
