# Supplementary material for: Electrochemical Performance of Metal-Free Carbon-Based Catalysts from Different Hydrothermal Carbonization Treatments for Oxygen Reduction Reaction
Source: Nanomaterials (Basel). 2024 Jan 12;14(2):173. doi: 10.3390/nano14020173 (PMC10820196; doi:10.3390/nano14020173)
Supplement: Supplementary file 1 [file nanomaterials-14-00173-s001.zip › nanomaterials-2795202-supplementary.pdf]

SUPPORTING INFORMATION

Electrochemical Performance of Metal-Free Carbon-Based Catalysts from Different Hydrothermal Carbonization Treatments for Oxygen Reduction Reaction

Aldo Girimonte [1], Andrea Stefani [2], Clara Mucci [1], Roberto Giovanardi [1], Andrea Marchetti [3], Massimo Innocenti [4,5], and Claudio Fontanesi [1,5]*

[1]University of Modena and Reggio Emilia, Department of Engineering, DIEF, via vivarelli 10, 41125 Modena, ITALY;
[2]University of Modena and Reggio Emilia, Department of Physics, FIM, via Campi 213, 41125 Modena, ITALY
[3]University of Modena and Reggio Emilia, Department of Chemical and Geological Science, DSCG, via Campi 103, 41125 Modena, ITALY
[4]University of Firenze, Department of Chemistry, "Ugo Schiff", via della Lastruccia 3, 50019 Sesto Fiorentino, ITALY
[5]National Interuniversity Consortium of Materials Science and Technology (INSTM), Via G. Giusti 9, 50121 Firenze, ITALY
***Correspondence: claudio.fontanesi@unimore.it

Impedance results

For the sake of comparison impedance spectra are here reported for **"dry"** nanospheres obtained by using the traditional hydrothermal procedure (T-HTC) as well as the MicroWave-ssisted HTC (MW-HTC) synthetic route. These data must be compared with impedance spectra reported in Figure 5 in the main manuscript.

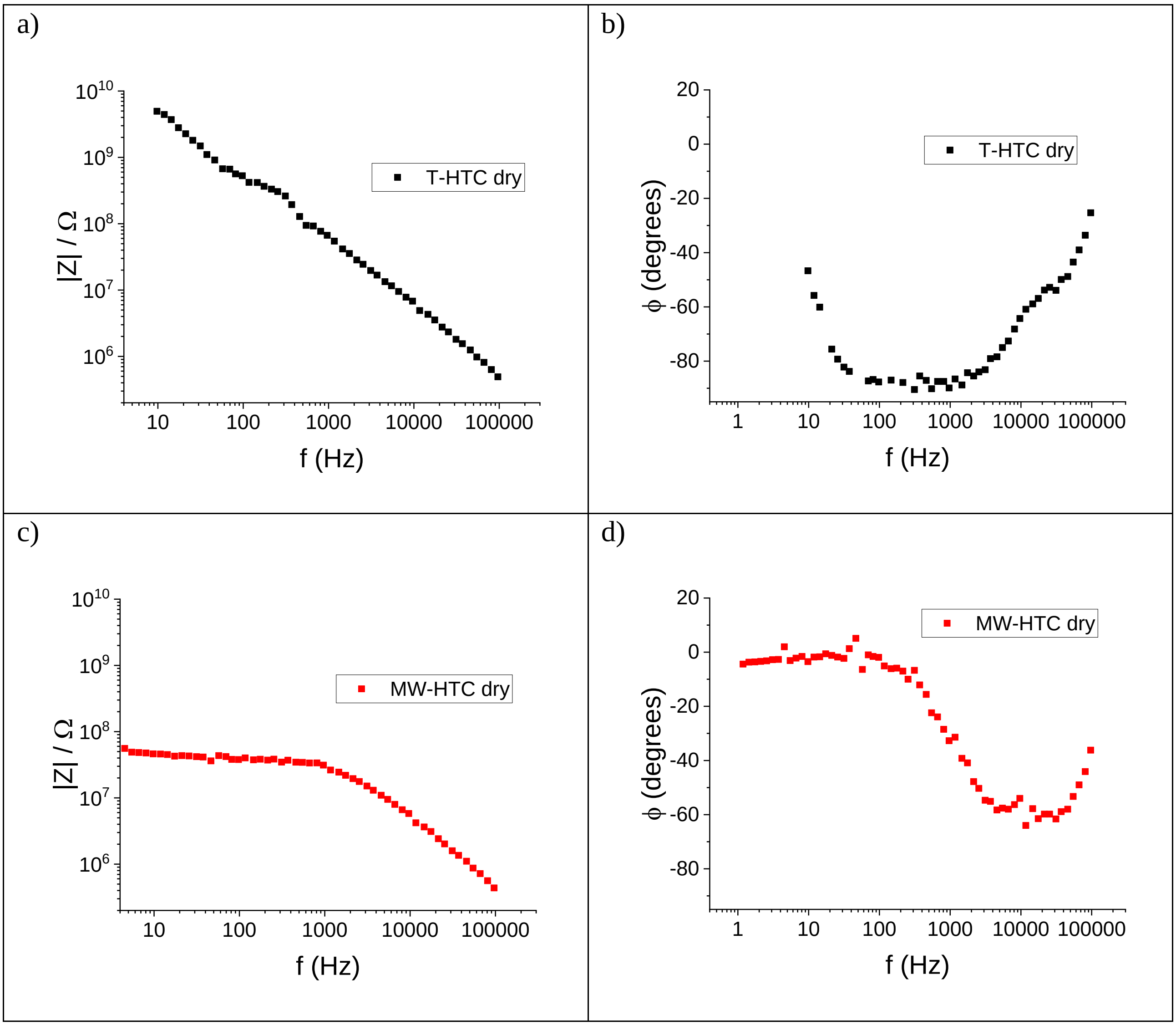

Figure S1. Impedace spectra recorded in the 100 kHz to 1 Hz frequecy range, Bode representation, are here reported for the glucose nanospheres in the dry state. a) sets out the impedance absolute value as a function of the frequency concerning the particles obtained via traditional hydrothermal synthetic route. b) sets out the phase as a function of the frequency. c) sets out the impedance absolute value as a function of the frequency concerning the particles obtained via microwave synthetic route. d) sets out the phase as a function of the frequency.

Remarkably the impedance of the T-HTC particles is higher than thst of the MW-HTC ones. The latter are characterized by a resistive electric behaviour in the low frequency limit (the phase in Figure S1d is close to zero and almost constant in the 1 to 1000 Hz frequency range. Interestingly, the impedance spectra of the ink, a situation which resembles more closely the solution environment, show a differet behaviour. The T-HTC particles are characterized by a lower value of impedance with respect to MW-HTC ones (compare Figure 5 of the main manuscript).
